# A Caprine Herpesvirus 1 Vaccine Adjuvanted with MF59™ Protects against Vaginal Infection and Interferes with the Establishment of Latency in Goats

**DOI:** 10.1371/journal.pone.0034913

**Published:** 2012-04-12

**Authors:** Mariarosaria Marinaro, Giovanni Rezza, Giuseppe Del Giudice, Valeriana Colao, Elvira Tarsitano, Michele Camero, Michele Losurdo, Canio Buonavoglia, Maria Tempesta

**Affiliations:** 1 Department of Infectious, Parasitic and Immune-mediated Diseases, Istituto Superiore di Sanità, Rome, Italy; 2 Research Center, Novartis Vaccines and Diagnostic, Siena, Italy; 3 Department of Veterinary Public Health, University of Bari, Bari, Italy; Southern Illinois University School of Medicine, United States of America

## Abstract

The immunogenicity and the efficacy of a beta-propiolactone-inactivated caprine herpesvirus 1 (CpHV-1) vaccine adjuvanted with MF59™ were tested in goats. Following two subcutaneous immunizations, goats developed high titers of CpHV-1-specific serum and vaginal IgG and high serum virus neutralization (VN) titers. Peripheral blood mononuclear cells (PBMC) stimulated *in vitro* with inactivated CpHV-1 produced high levels of soluble IFN-gamma and exhibited high frequencies of IFN-gamma producing cells while soluble IL-4 was undetectable. On the other hand, control goats receiving the inactivated CpHV-1 vaccine without adjuvant produced only low serum antibody responses. A vaginal challenge with virulent CpHV-1 was performed in all vaccinated goats and in naïve goats to assess the efficacy of the two vaccines. Vaginal disease was not detected in goats vaccinated with inactivated CpHV-1 plus MF59™ and these animals had undetectable levels of infectious challenge virus in their vaginal washes. Goats vaccinated with inactivated CpHV-1 in the absence of adjuvant exhibited a less severe disease when compared to naïve goats but shed titers of challenge virus that were similar to those of naïve goats. Detection and quantitation of latent CpHV-1 DNA in sacral ganglia in challenged goats revealed that the inactivated CpHV-1 plus MF59™ vaccine was able to significantly reduce the latent viral load when compared either to the naïve goats or to the goats vaccinated with inactivated CpHV-1 in the absence of adjuvant. Thus, a vaccine composed of inactivated CpHV-1 plus MF59™ as adjuvant was strongly immunogenic and induced effective immunity against vaginal CpHV-1 infection in goats.

## Introduction

Caprine herpesvirus 1 (CpHV-1) is an *Alphaherpesvirus* [1] responsible for lethal systemic infections in 1- to 2-week-old kids [2], [3] and for mild to subclinical infections in adult goats [4], [5]. Clinical manifestations in adult goats involve the respiratory or the reproductive tract [6] depending on the site of virus entry although CpHV-1 infects preferentially the genital mucosa. Following primary genital infection, the virus replicates in the mucosal epithelium and spreads to sacral ganglia to establish latency [7]. Genital CpHV-1 infections are characterized by painful erythematous-oedematous lesions evolving into vesicles and ulcers healing in two weeks [8]; balanoposthitis, vaginitis, infertility or abortion are often observed during primary or recurrent infections. Although the CpHV-1 infection is distributed worldwide and major economical losses occur in Mediterranean countries, no vaccines are commercially available. An ideal vaccine against CpHV-1 should prevent primary infection and replication in the vaginal mucosa and should interfere with the establishment of latency; in fact, reactivation of latent virus and mucosal shedding are responsible for CpHV-1 transmission to other animals in the same flock and to newborns. Interestingly, CpHV-1 shares several biological features with human HSV-2, such as, the tropism for the vaginal epithelium, the type of genital lesions and the establishment of latency in sacral ganglia [7], [9], [10].

Experimental studies in goats have shown that parenteral immunization with inactivated CpHV-1 plus Montanide ISA™ 740 or vaginal immunization with inactivated CpHV-1 plus LTK63, provide partial protection against a vaginal challenge with virulent CpHV-1 [11], [12]. In addition, intranasal vaccination with a live attenuated gE negative Bovine herpesvirus 1 (BoHV-1) vaccine was shown to confer partial cross-reactive protection to goats challenged vaginally with CpHV-1 [13]. Collectively, the above studies have suggested that either virus neutralization (VN) activity in serum or CpHV-1-specific vaginal IgA contribute to protection although the precise role played by antibody responses to protection against vaginal CpHV-1 infection needs to be specifically addressed. In addition, the role played by cell-mediated immune responses in controlling CpHV-1 infection and reactivation remains unknown.

The present study was undertaken to determine if the use of a potent adjuvant could augment the immunogenicity and the protective efficacy of an inactivated CpHV-1 vaccine. To this end, goats were subcutaneously immunized with a beta-propiolactone-inactivated CpHV-1 vaccine and MF59™ as adjuvant. The oil-in-water emulsion MF59™ was employed since it is licensed for human use with an influenza vaccine since 1997 [14], it has a large safety database [15], [16] and it is known to induce both antibody and cell-mediated immune responses in various preclinical models and humans [14], [17] although it has never been tested for efficacy in ruminants.

The results presented herein provide the first evidence that the addition of the MF59™ adjuvant greatly enhances the immunogenicity and protection afforded by an inactivated CpHV-1 vaccine.

## Materials and Methods

### Ethics Statement

The experiments were approved by the Italian Ministry of Health (Prot. n. 2174/07) and were carried out at the University of Bari according to the National Guide for Care and Use of Experimental Animals.

### Animals

Twenty-eight female goats (18 months-2 years) of mixed breed were employed in the study. Before vaccination the goats were tested to insure that they were negative for both serum VN antibodies, serum CpHV-1-specific IgG and vaginal CpHV-1-specific IgG [18].

### Virus, inactivated vaccine and virus challenge

The CpHV-1 Ba.1 strain [19] was used throughout the study to prepare: i) the inactivated vaccine; ii) the antigen for the ELISA and iii) the inoculum for the vaginal challenge. Briefly, the virus stock was obtained by infecting Madin Darby Bovine Kidney cells (MDBK; ATCC LGC Standards, Milan, Italy) grown in DMEM (Lonza, Walkersvillee, USA). The viral titer was 10^6.5^ 50% tissue culture infectious doses (TCID_50_/50 µl). The viral suspension was tested and found to be free from bacterial and fungal contamination. For the vaccine preparation, the virus was inactivated with beta-propiolactone as described by Tempesta et al. [11]. The total amount of proteins in the inactivated CpHV-1 suspension was 180±10 µg/ml. The vaginal challenge was performed by pipetting four milliliters of virulent CpHV-1 suspension (10^5^ TCID_50_/50 µl) into the vaginal lumen of naïve animals or vaccinated animals.

### Vaccination

The adjuvant employed in the study was the oil-in-water emulsion MF59™ (Novartis Vaccines and Diagnostics, Siena, Italy). The twenty-eight goats received either 2 ml of inactivated CpHV-1 (10^6.5^ TCID_50_/50 µl) plus 2 ml of sterile saline solution or received 2 ml of inactivated CpHV-1 emulsified with 2 ml of MF59™ or were left unvaccinated (these animals served as naïve controls in challenge studies). Goats were employed to perform four independent experiments conducted as follows: experiment n.1, n = 4 goats (one goat received inactivated CpHV-1 only; two goats received inactivated CpHV-1 plus MF59™; one goat remained unvaccinated); experiment n.2, n = 6 goats (one goat received inactivated CpHV-1 only; four goats received inactivated CpHV-1 plus MF59™; one goat remained unvaccinated); experiment n.3, n = 9 goats (three goats received inactivated CpHV-1 only; three goats received inactivated CpHV-1 plus MF59™; three goats remained unvaccinated); experiment n.4, n = 9 goats (three goats received inactivated CpHV-1 only; three goats received inactivated CpHV-1 plus MF59™; three goats remained unvaccinated). After preparation, vaccines were immediately injected subcutaneously in the neck. All goats were vaccinated with two doses of vaccine (one dose on day 0 and one dose on day 10). On close inspection of the injection site there was no evidence of local reaction, granulomas or abscesses in goats vaccinated with inactivated CpHV-1 with or without the adjuvant MF59™. No systemic adverse reactions were observed in any goat included in the study.

### Collection of serum samples, vaginal washes and peripheral blood mononuclear cells

Blood samples were aseptically obtained from the jugular vein before each immunization (days 0 and 10) and 10 days after the second immunization (day 20). Heparinized tubes were employed to collect whole blood for cellular analyses. Serum was obtained after centrifugation of non-heparinized glass tubes at 2,000 rpm for 10 min (Beckman microfuge, Fullerton, USA). Vaginal samples were collected by flushing the vaginal lumen with a sterile pipette containing 2 ml of sterile PBS; the samples were then centrifuged at 2,000 rpm for 10 min and supernatants were collected. Sera and vaginal washes were stored at −80°C until tested.

### Virus neutralization assay

Virus neutralization (VN) assays were performed as described elsewhere [11], [18]. Briefly, sera were heat-inactivated at 56°C for 30 min and serial 2-fold dilutions starting from 1∶2 (of each individual sample) were mixed with 100 TCID_50_ of CpHV-1 Ba.1 strain in 96-well microtiter plates (Corning Inc., NY, USA). The plates were kept at room temperature for 45 min, and then 20,000 MDBK cells were added to each well. After incubation for 3 days at 37°C with 5% of CO_2_, the endpoint titers were determined using the Spearman-Karber method and expressed as the highest serum dilution able to neutralize the cytopathic effect.

### ELISA for measurement of IgG, IgG subclasses and IgA

The ELISA tests employed to determine the titers of CpHV-1-specific IgG or IgA in serum and vaginal washes and the titers of CpHV-1-specific serum IgG1/IgG2 subclasses are described elsewhere [18], [20]. Briefly, 96-well polystyrene plates (Nunc, Roskilde, Denmark) were coated with CpHV-1 diluted in carbonate buffer and incubated overnight at 4°C on a shaker. After blocking and washing steps, serial 2-fold dilutions of individual sera or vaginal washes were added to duplicate wells. After incubation overnight at 4°C, plates were washed and HRP-conjugated rabbit anti-goat IgG (Bethyl, Montgomery, USA) or HRP-conjugated rabbit anti-goat IgA (Bethyl) or HRP-conjugated sheep anti-bovine IgG1 (Bethyl) or HRP-conjugated sheep anti-bovine IgG2 (Bethyl) were added to the wells and incubated overnight at 4°C. After final washings and addition of ABTS, the colorimetric reaction was measured at 405 nm with an ELISA plate reader (Biorad, Hercules, USA). The O.D. values were recorded and individual readings were reported by subtracting the O.D. values of negative control sera to the individual O.D. values. The antibody titers were determined as the reciprocal of the highest sample dilution exhibiting an O.D. value of 0.2 units above the O.D. of negative control sera.

### In vitro IFN-gamma and IL-4 measurements

Peripheral blood mononuclear cells (PBMC) were isolated from heparinized blood samples using the standard density-gradient separation procedure (Lympholyte, CEDARLANE laboratories Ltd., Burlington, NC, USA) and washed twice with sterile HBSS. PBMC at 2×10^6^ viable cells/ml were incubated for 5 days at 37°C with 5% CO_2_ in complete medium (RPMI 1640 supplemented with 100 U/ml penicillin, 100 µg/ml streptomycin, 2 mM l-glutamine, 10% heat-inactivated FBS) for determination of cytokine secretion. PBMC cultures were stimulated with inactivated CpHV-1 (10 µg/ml) since previous studies [21] showed that optimal cytokine secretion was induced with that amount of antigen; MDBK cell lysates without virus did not stimulate cytokine secretion thus excluding any cell-specific cytokine response. Cultures left unstimulated served as negative controls. In each experiment, parallel cultures were set up with PBMC isolated from goats: i) vaccinated with inactivated CpHV-1 only; ii) vaccinated with inactivated CpHV-1 plus MF59™; iii) naïve. The levels of soluble IL-4 in culture supernatants were measured by using a commercially available ELISA kit (Thermo Scientific) shown to be able to detect caprine IL-4 [21] and the test was performed according to the manufacturer's instructions. The levels of soluble IFN-gamma in culture supernatants were determined by using a commercially available ELISA kit (Bovigam, Prionics, Victoria, Australia) with a modification since quantification was achieved by using bovine recombinant IFN-gamma (Thermo Scientific) as a standard. The sensitivity of the IL-4 and IFN-gamma kits was 5 pg/ml. For the measurement of IFN-gamma spot-forming cells (IFN-gamma SFC) a commercially available ELISPOT kit specific for bovine IFN-gamma and cross-reactive with caprine IFN-gamma [21] was employed and the test was performed according to the manufacturer's instructions. In particular, PBMC suspensions were directly cultured onto nitrocellulose-well plates included in the kit (Thermo Scientific, Rockford, IL, USA) and IFN-gamma SFC were counted with the aid of a dissecting microscope (Leica MS5, Leica Microsystems Srl, Milan, Italy).

### Titration of viral shedding and measurement of clinical scores after vaginal challenge

All animals (vaccinated and unvaccinated) were subjected to vaginal challenge with 4 ml of virulent CpHV-1 (10^5^ TCID_50_/50 µl) and were kept under observation for 14 days. The challenge was performed two weeks following the second immunization (i.e., day 25 post first immunization) in vaccinated animals. Following the challenge, viral shedding was measured according to Tempesta et al. [12]. Briefly, vaginal swabs were collected daily and placed in 1.5 ml of DMEM then were centrifuged at 10,000 rpm for 5 min. The supernatant was collected (0.9 ml), treated with 0.1 ml of an antibiotic solution (5,000 IU/ml penicillin, 2,500 µg/ml streptomycin and 10 µg/ml amphotericin B) and incubated for 30 min at room temperature. Each sample was serially diluted (10-fold), and inoculated (in quadruplicate) onto MDBK cells placed in 96-well microtiter plates (Corning). Plates were incubated for 3 days at 37°C with 5% CO_2_ and the virus titer was measured as previously described [12]. After the challenge, goats were also examined daily to evaluate the severity of the CpHV-1 infection. The body temperature was measured, and general and local clinical signs were recorded (i.e., hyperemia, edema, lesions, pain). A cumulative clinical score was determined in each animal by grading the clinical signs as follows: 0, absent; 1, mild; 2, moderate; and 3, severe. Temperature increments above normal (the normal body temperature ranged from 38.2 to 38.6°C) were graded as follows: >0.5–1°C = 1; >1.1–1.5°C = 2; >1.5°C = 3.

### Real-time PCR

A real-time PCR assay was employed to detect and quantitate CpHV-1 genomic DNA in vaginal swabs (collected daily for 14 days after the challenge) and sacral ganglia (excised one month after challenge). The assay was conducted as previously reported [22]. Briefly, samples (i.e., 200 µl of the swab or 25 mg of each ganglion) were processed to extract the DNA (QIAGEN S.p.A. Italy). Primers, probes, and CpHV-1 DNA standards employed in the real-time PCR assay were designed to target a conserved region of the gC gene of CpHV-1 Ba.1 strain [22]. An internal control (canine parvovirus type 2 DNA) was included in each assay to exclude any loss of DNA during the extraction and amplification steps. The analytical performance of the real-time PCR assay employed in this study was comparable to that reported previously [22], [23] while the detection limit of the assay was 5×10^1^ standard DNA copies/10 µl of template.

### Statistical analysis

Data are expressed as arithmetic mean ± SD of the: i) log_2_ transformed VN titers; ii) log2 transformed serum IgG/vaginal IgG/serum IgG subclass titers; iii) soluble IFN-gamma levels; iv) numbers of IFN-gamma SFC; v) log_10_ transformed viral shedding titers; vi) numbers of CpHV-1 genomes in vaginal swabs; vi) clinical scores.

Data on latency in sacral ganglia are expressed as arithmetic mean ± SEM of the log_10_ transformed number of CpHV-1 genomes.

Data recorded from day 0 to day 14 post challenge (i.e, vaginal CpHV-1 shedding titers, number of CpHV-1 genomes in vaginal swabs and clinical scores) were used to calculate the Area Under Curve (AUC).

Data were analyzed by the one-way ANOVA test followed by the Tukey's post-hoc test for multiple comparisons.

The difference between log_2_ serum anti-CpHV-1 IgG1 and IgG2 titers (within the same vaccine group) was analyzed by the Students' *t*-test.

The software *R* (version 2.8.1) was employed to perform all tests. A *p* value less than 0.05 was considered significant.

## Results

### CpHV-1-specific serum IgG and VN antibodies induced after vaccination with inactivated CpHV-1 plus MF59™

To study the immunogenicity of the inactivated CpHV-1 plus MF59™ vaccine, VN antibodies and IgG specific to CpHV-1 were measured in serum. Both parameters were measured in sera drawn before vaccination (day 0), ten days after the first dose of vaccine (day 10) and ten days after the second dose of vaccine (day 20). In order to evaluate the adjuvant activity of MF59™, goats vaccinated with inactivated CpHV-1 only were used as controls while naïve goats served as background controls. It should be noted that day 0 samples from all vaccinated and naïve goats had undetectable levels of: i) VN serum antibodies; ii) CpHV-1-specific serum IgG and iii) CpHV-1-specific vaginal IgG (as these were inclusion criteria for the current study).

**Figure 1:**
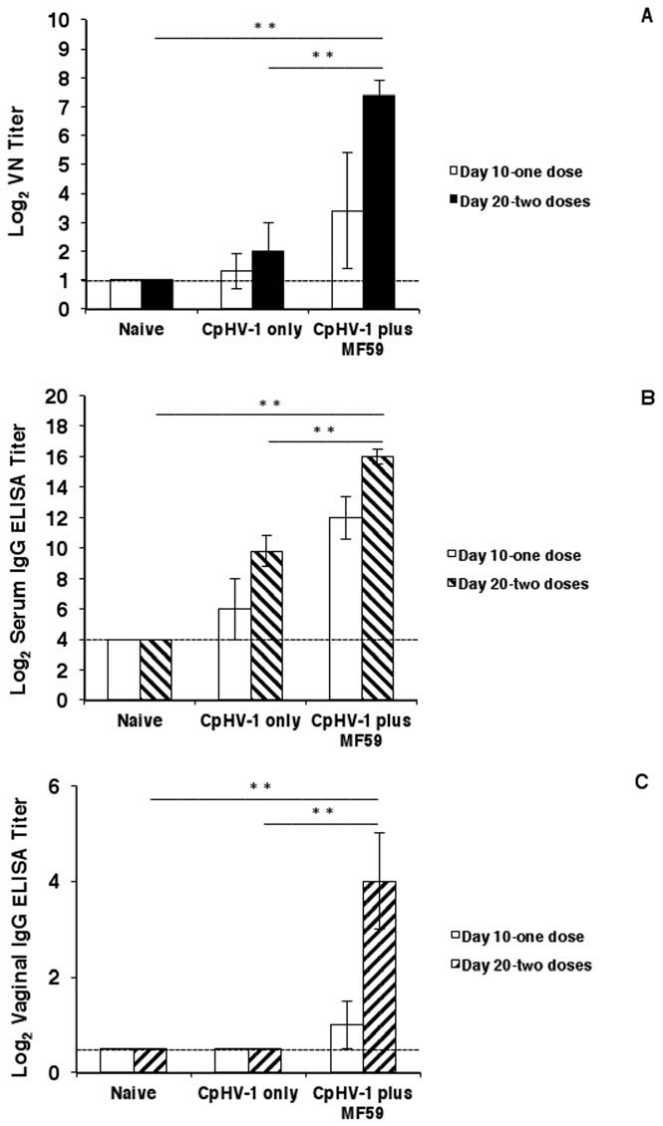
Serum and vaginal antibody responses in goats subcutaneously vaccinated with inactivated CpHV-1 plus MF59™. Vaccines were given on day 0 and day 10. Sera and vaginal washes were collected on day 10 (white histograms) and day 20 (black or dashed histograms) to determine serum VN titers (A) or ELISA serum IgG titers (B) or ELISA vaginal IgG titers (C). Data are expressed as arithmetic mean ± SD. Data reported are cumulative from 4 independent experiments performed as described in Materials and Methods with a total of 8 goats in the naïve group, a total of 8 goats in the inactivated CpHV-1 only vaccinated group and a total of 12 goats in the inactivated CpHV-1 plus MF59™ vaccinated group. A double asterisk (**) denotes probability, p, <0.001 that the VN titers (A) or the serum IgG titers (B) or the vaginal IgG titers (C) in goats vaccinated with inactivated CpHV-1 plus MF59™ were equivalent to those of naïve goats on day 20 post immunization. The same probability, p, <0.001 was observed when VN titers (A), serum IgG titers (B) and vaginal IgG titers (C) in goats vaccinated with inactivated CpHV-1 plus MF59™ were compared to those of goats vaccinated with inactivated CpHV-1 only on day 20 post immunization. Statistical differences were calculated by the one-way ANOVA test followed by the Tukey's post-hoc test.

As shown in figure 1A, after the first dose of the vaccines (i.e., day 10 sera), goats exhibited a variable VN antibody response to CpHV-1. In particular, in goats vaccinated with inactivated CpHV-1 only, the average VN titers were slightly above those of naïve goats (<log_2_ 1 in naïve goats; log_2_ 1.3±0.6 in goats vaccinated with inactivated CpHV-1 only) while goats vaccinated with inactivated CpHV-1 plus MF59™ showed average VN titers (log_2_ 3.4±2) which were 4- to 8-fold higher than those of naïve goats and were 4-fold higher than those of goats vaccinated with inactivated CpHV-1 only.

After the administration of the second dose of the vaccine i.e., on day 20 post immunization (Fig. 1A), VN titers increased in goats immunized with CpHV-1-only but reached average titers (log_2_ 2±1) that were only 2-fold higher than those of naïve animals. In contrast, goats vaccinated with inactivated CpHV-1 plus MF59™ developed significantly elevated VN titers (log_2_ 7.4±0.5) on day 20 which were approximately 128-fold higher than those of naïve goats and 64-fold higher than those of goats vaccinated with inactivated CpHV-1 only.

Figure 1B shows that after the first dose of the vaccines, i.e., on day 10 post immunization, the titers of CpHV-1-specific serum IgG in goats vaccinated with inactivated CpHV-1 only (log_2_ 6±2), were 4-fold above those of naïve goats (<log_2_ 5) while goats vaccinated with inactivated CpHV-1 plus MF59™ developed IgG titers (log_2_ 12±1.4) that were 256-fold above those of naïve goats. The second dose of the vaccines boosted the serum IgG responses in all goats although goats vaccinated with inactivated CpHV-1 only exhibited average IgG titers (log_2_ 9.8±1) on day 20 post immunization that were 64-fold higher than those of naïve goats while goats vaccinated with inactivated CpHV-1 plus MF59™ developed significant titers of serum IgG specific to CpHV-1 (log_2_ 16±0.5) which were approximately 64-fold higher than titers measured in goats vaccinated with inactivated CpHV-1 only and were at least 4096-fold higher than those of naïve goats.

**Figure 2:**
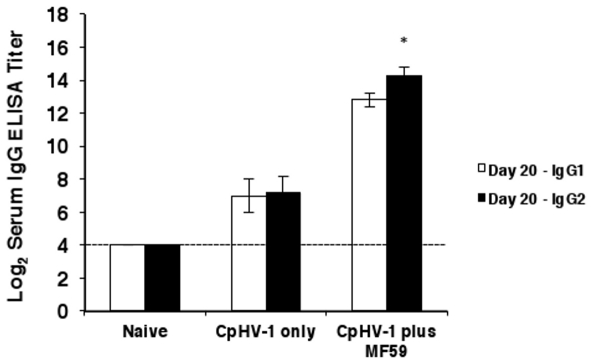
Serum IgG subclass responses in goats subcutaneously vaccinated with inactivated CpHV-1 plus MF59™. Vaccines were given on day 0 and day 10. Sera were collected on day 20 to determine CpHV-1-specific IgG1 titers (white histograms) and CpHV-1-specific IgG2 titers (black histograms) by ELISA. Data are expressed as arithmetic mean ± SD. Data reported are cumulative from 4 independent experiments performed as described in Materials and Methods with a total of 8 goats in the naïve group, a total of 8 goats in the inactivated CpHV-1 only vaccinated group and a total of 12 goats in the inactivated CpHV-1 plus MF59™ vaccinated group. A single asterisk (*) denotes probability, p, <0.05 that the serum IgG1 titers are equivalent to the serum IgG2 titers in goats vaccinated with inactivated CpHV-1 plus MF59™. The Students' *t*-test was employed to study statistical differences.

Titers of IgG1 and IgG2 specific to CpHV-1 were measured in sera from all vaccinated goats after the second immunization i.e., at day 20, to study the relative contribution of IgG subclasses to the observed IgG response [20], [24]. Goats vaccinated with inactivated CpHV-1 only, produced low titers of both IgG1 and IgG2 subclasses (log_2_ 7±1.1 and log_2_ 7.2±1.1, respectively) (Fig. 2) which were an average 8-fold higher than background levels in naïve goats (<log_2_ 5). In contrast, goats vaccinated with inactivated CpHV-1 plus MF59™ exhibited titers of serum IgG1 (log_2_ 12.8±0.4) that were an average 256-fold higher than those of naïve goats and titers of serum IgG2 (log_2_ 14.3±0.5) that were an average 512-fold higher than those of naïve goats. In addition, in goats vaccinated with CpHV-1 plus MF59™ titers of serum IgG2 were significantly higher than those of IgG1 (Fig. 2).

In all vaccinated goats the levels of CpHV-1-specific serum IgA were also studied by ELISA and they were undetectable at all time points tested (data not shown).

### Subcutaneous vaccination with inactivated CpHV-1 plus MF59™ results in CpHV-1-specific vaginal IgG

The systemic immune system may contribute to local protection in the genital mucosa with antibodies transudating from serum into vaginal secretions [25]–[28]. Since the inactivated CpHV-1 plus MF59™ vaccine induced high serum titers of antigen-specific IgG and high VN titers, the same parameters were measured in vaginal washes collected from vaccinated goats. After administration of the first vaccine dose (i.e., samples collected on day 10), none of the goats vaccinated with CpHV-1 only exhibited detectable CpHV-1-specific vaginal IgG (<log_2_ 1; Fig. 1C) while animals vaccinated with inactivated CpHV-1 plus MF59™ exhibited low titers of vaginal IgG (log_2_ 1±0.5) which were an average 2-fold higher than those of naïve goats (<log_2_ 1; Fig. 1C). Administration of the second vaccine dose (i.e., samples collected on day 20) resulted in significantly elevated titers of CpHV-1-specific vaginal IgG (log_2_ 4±1) in goats vaccinated with inactivated CpHV-1 plus MF59™ and these antibody titers were an average 16-fold higher than those of naïve goats (Fig. 1C). On day 20 post immunization, CpHV-1-specific vaginal IgG remained undetectable in goats vaccinated with inactivated CpHV-1 only.

It should be noted that the levels of vaginal IgG in the three groups of goats were different despite the presence of similar levels of albumin in vaginal samples (data not shown) suggesting that vaginal IgG in goats vaccinated with inactivated CpHV-1 plus MF59™ reflected the higher levels of serum IgG in this group of animals rather than resulting from an augmented mucosal permeability.

The level of VN activity in vaginal washes from all vaccinated goats was undetectable even after the second immunization (i.e., day 20) and this was likely due to the dilution of vaginal antibodies during the collection of the sample [29]. No CpHV-1-specific vaginal IgA were detected in any animal in response to the vaccines at any time (data not shown).

### Production of IFN-gamma by antigen-specific PBMC from goats vaccinated with inactivated CpHV-1 plus MF59™

**Figure 3:**
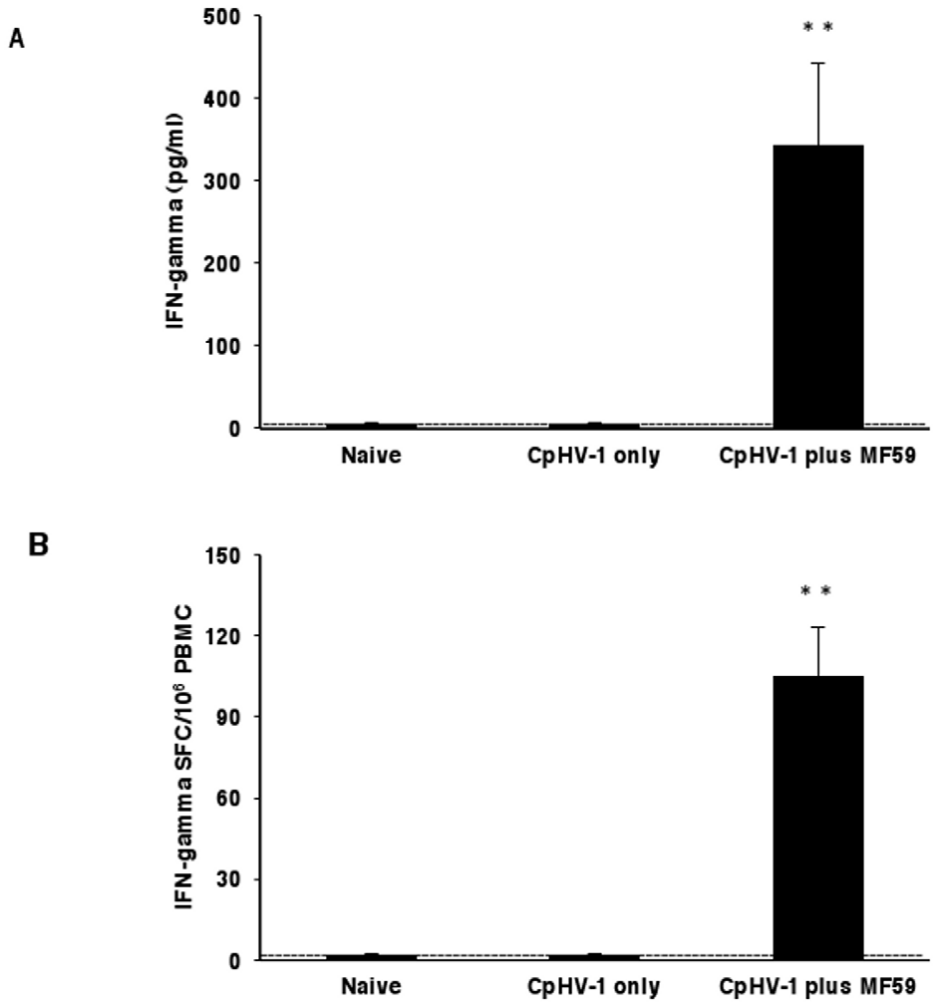
IFN-gamma production by PBMC isolated from goats subcutaneously vaccinated with inactivated CpHV-1 and MF59™. Vaccines were given on day 0 and day 10. Blood was drawn on day 20 and PBMC were isolated and stimulated *in vitro* as described in Materials and Methods. Soluble IFN-gamma in culture supernatants was measured by ELISA (A) while frequencies of IFN-gamma Spot Forming Cells (SFC) were measured by ELISPOT (B). Data (arithmetic mean ± SD) are cumulative from 4 independent experiments performed as described in Materials and Methods with a total of 8 goats in the naïve group, a total of 8 goats in the inactivated CpHV-1 only vaccinated group and a total of 12 goats in the inactivated CpHV-1 plus MF59™ vaccinated group. A double asterisk (**) denotes probability, p, <0.001 that the soluble IFN-gamma levels (A) or the IFN-gamma SFC (B) in goats vaccinated with inactivated CpHV-1 plus MF59™ were equivalent to those of naïve goats. The same probability, p, <0.001 was observed when soluble IFN-gamma levels (A) or IFN-gamma SFC (B) in goats vaccinated with inactivated CpHV-1 plus MF59™ were compared to those of goats vaccinated with inactivated CpHV-1 only. Statistical differences were calculated by the one-way ANOVA test followed by the Tukey's post-hoc test.

The profile of IFN-gamma and IL-4 secretion by PBMC isolated from vaccinated goats (following the second immunization i.e., on day 20) was studied in culture supernatants after CpHV-1 stimulation. As shown in Fig. 3A, PBMC isolated from goats vaccinated with inactivated CpHV-1 only and from naïve goats did not produce detectable levels of IFN-gamma upon antigen-stimulation *in vitro*. Goats vaccinated with inactivated CpHV-1 plus MF59™ exhibited significant levels of soluble IFN-gamma (343±101 pg/ml) in response to *in vitro* antigen stimulation and these levels were approximately 150-fold higher than those of either naïve goats or goats vaccinated with inactivated CpHV-1 only (<5 pg/ml). In the same cultures, IL-4 was always undetectable in response to CpHV-1 stimulation (data not shown). Mitogen-stimulated PBMC cultures from all vaccinated goats and from naïve goats, secreted similar amounts of both IFN-gamma and IL-4 (data not shown) indicating that the ability to produce IFN-gamma or IL-4 was intact and comparable in all goats.

The frequencies of CpHV-1-specific IFN-gamma producing cells were measured by ELISPOT (Fig. 3B) and PBMC from goats vaccinated with inactivated CpHV-1 plus MF59™ developed significantly more IFN-gamma producing cells (105±18/10^6^ PBMC) relative either to naïve goats or to goats vaccinated with inactivated CpHV-1 only (<1 IFN-gamma SFC/10^6^ PBMC).

### An inactivated CpHV-1 plus MF59™ vaccine reduces viral replication and protects against vaginal diseases in goats vaginally challenged with virulent CpHV-1

**Figure 4:**
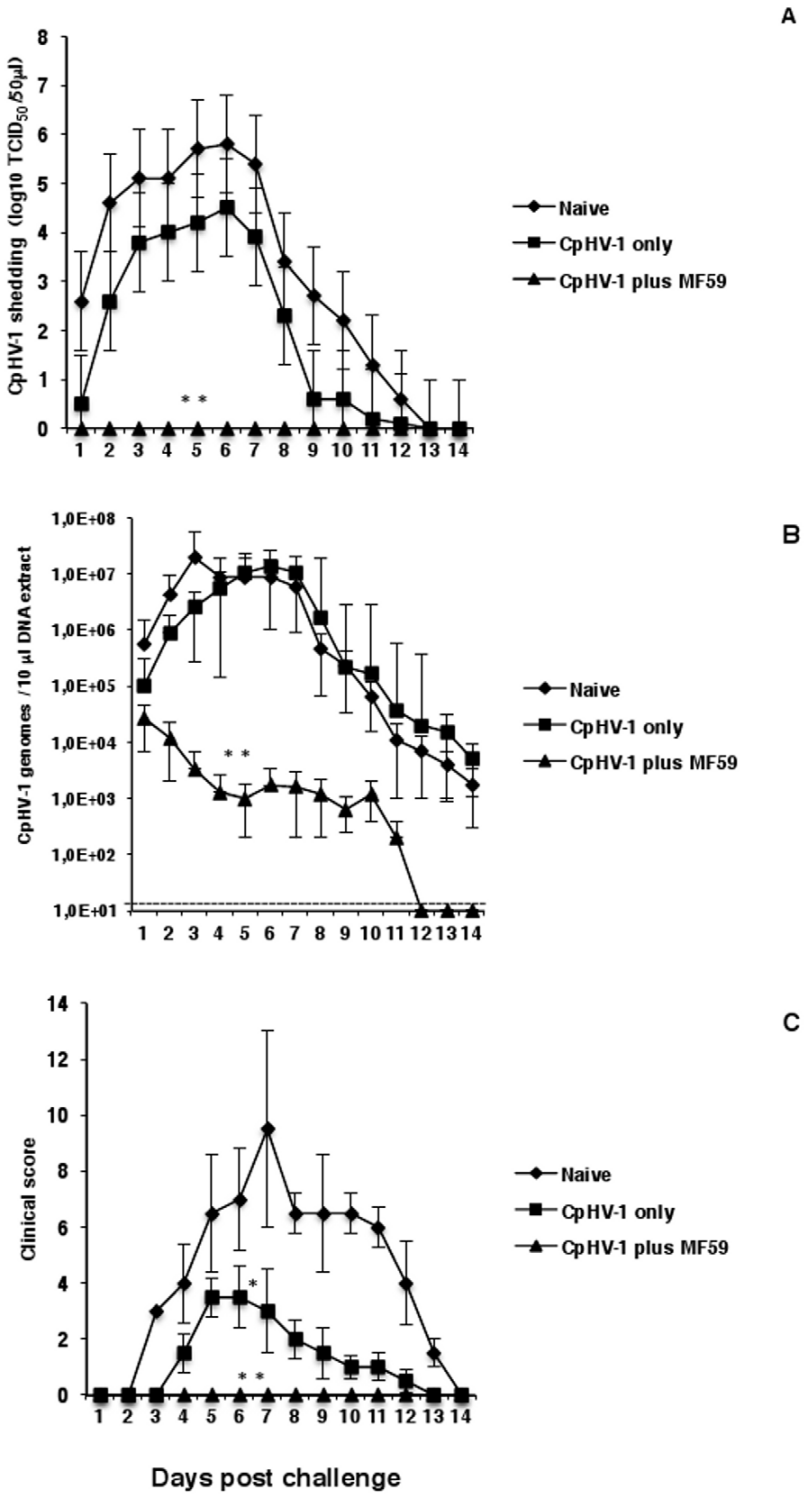
Vaginal viral shedding and clinical scores in goats vaccinated with inactivated CpHV-1 and challenged vaginally with virulent CpHV-1. Vaccines were given on day 0 and day 10. All goats were challenged vaginally two weeks following the second vaccination as described in Materials and Methods with 4 ml of virulent CpHV-1 suspension (10^5^ TCID_50_/50 µl). Naïve goats were included as unvaccinated controls. Goats were monitored daily for 14 days post-challenge and vaginal swabs were collected daily and employed to quantitate the viral shedding. Titration of the infectious virus in vaginal swabs was achieved by measuring the cytopathic effect of serial dilutions of the sample in MDBK cultures (A) while the number of CpHV-1 genomes in vaginal swabs was measured by real-time PCR (B). Clinical scores were also recorded daily following the challenge (C). Data (arithmetic mean ± SD) are cumulative from 4 independent experiments performed as described in Materials and Methods with a total of 8 goats in the naïve group, a total of 8 goats in the inactivated CpHV-1 only vaccinated group and a total of 12 goats in the inactivated CpHV-1 plus MF59™ vaccinated group. In (A), (B) and (C) the double asterisk (**) denotes probability, p, <0.001 that the CpHV-1 shedding titers (A) or the number of CpHV-1 genomes (B) or the clinical scores (C) in goats vaccinated with inactivated CpHV-1 plus MF59™ were equivalent to those of naïve goats. The same probability, p, <0.001 was observed when the CpHV-1 shedding titers (A) or the number of CpHV-1 genomes (B) or the clinical scores (C) in goats vaccinated with inactivated CpHV-1 plus MF59™ were compared to those of goats vaccinated with inactivated CpHV-1 only. In (C) the single asterisk denotes probability, p, <0.05 that the clinical scores in goats vaccinated with inactivated CpHV-1 only were equivalent to those of naïve goats. Data depicted in (A), (B) and (C), were used to calculate the AUC in order to perform statistical analyses. Statistical differences were calculated by the one-way ANOVA test followed by the Tukey's post-hoc test.

To determine the efficacy of the inactivated CpHV-1 plus MF59™ vaccine, all vaccinated goats were vaginally infected with 4 ml of virulent CpHV-1 suspension (10^5^ TCID_50_/50 µl) two weeks after the second immunization and were monitored daily, for 14 days, in order to record the severity of disease and the vaginal virus shedding. The kinetics of these parameters was determined and the area under curve (AUC) was calculated in each goat for statistical comparisons. Naïve goats were vaginally infected and used as unvaccinated controls. Figure 4A shows that from day 1 to day 14 post challenge, goats immunized with inactivated CpHV-1 only and naïve goats shed titers of challenge virus (measured by the standard cell-culture method) that were not significantly different. In particular, from day 1 to day 6 post challenge, the above groups of goats showed a similar trend of active local virus replication as they shed increasing titers of challenge virus that reached maximal levels by day 6. (log_10_ 2.6±1.4 on day 1 and log_10_ 5.8±1.1 on day 6 in naïve goats; log_10_ 0.5±0.3 on day 1 and log_10_ 4.5±1.5 on day 6 in goats vaccinated with inactivated CpHV-1 only). In the same groups of goats, the shedding of challenge virus started to decrease from day 7 post challenge to reach undetectable levels on days 13–14. On the other hand, at all time points post challenge, vaginal CpHV-1 shedding was not detected by the standard cell-culture test in any goat vaccinated with inactivated CpHV-1 plus MF59™ (Fig. 4A). The shedding of challenge virus in goats vaccinated with inactivated CpHV-1 plus MF59™ was statistically different from that of naïve goats and from that of goats vaccinated with inactivated CpHV-1 only.

A parallel real-time PCR assay was performed with DNA extracted from vaginal swabs collected daily from all challenged goats in order to detect and quantitate the number of CpHV-1 genomes. Figure 4B shows that, from day 1 to day 14 post challenge, the number of CpHV-1 genomes detected in vaginal swabs from goats immunized with CpHV-1 only, did not differ significantly from that of naïve goats. In particular, from day 1 to day 6 post challenge both the goats vaccinated with inactivated CpHV-1 only and the naïve goats exhibited a trend of active local virus replication with numbers of CpHV-1 genomes increasing from day 1 to day 6 post challenge. CpHV-1 genomes peaked at 2×10^7^±3.7×10^7^ on day 3 post challenge for naïve goats while the number of CpHV-1 genomes peaked at 1.4×10^7^±1.3×10^7^ on day 6 post challenge for goats vaccinated with inactivated CpHV-1 only. In these same groups of goats, the number of CpHV-1 genomes progressively decreased from day 7 to day 14 post challenge and reached levels on day 14 that were approximately 300-fold lower than those measured on day 1 post challenge in naïve goats and approximately 20-fold lower than those measured on day 1 post challenge in goats vaccinated with inactivated CpHV-1 only. It was interesting to note that, in vaginal samples obtained from goats vaccinated with inactivated CpHV-1 only and from naïve goats, the CpHV-1 DNA remained detectable on day 13 and on day 14 post challenge (Fig. 4B) when the titration of infectious virus by the cell-culture test gave negative results in the majority of goats (Fig. 4A).

A different vaginal CpHV-1 genome profile was observed in goats vaccinated with inactivated with CpHV-1 plus MF59™ (Fig. 4B). In fact, a decreasing number of CpHV-1 genomes was detected from day 1 to day 11 post challenge and from day 12 post challenge the CpHV-1 genomes became undetectable in vaginal swabs from this group of vaccinated goats (Fig. 4B). In addition, the number of vaginal CpHV-1 genomes measured from day 1 to day 14 post challenge was significantly different from that of naïve goats as well as from that of goats immunized with inactivated CpHV-1 only. It is remarkable that, in goats vaccinated with inactivated CpHV-1 plus MF59™, the infectious challenge virus was undetectable by the standard cell-culture test from day 1 to day 14 post challenge (Fig. 4A) and this was probably due to neutralization of the virus (whose genome was detected) by the antigen-specific vaginal antibodies (Fig. 1C). In addition, the number of CpHV-1 genomes detected on day 1 post challenge in vaginal swabs from goats vaccinated with inactivated CpHV-1 plus MF59™ (2.7×10^4^±2.0×10^4^) was approximately 20-fold lower than that measured in swabs from naive goats (5.7×10^5^±4.8×10^5^) and approximately 4-fold lower than that measured in goats vaccinated with inactivated CpHV-1 only (1.1×10^5^±9.7×10^4^) (Fig. 4B). Since all the animals were experimentally infected with an identical amount of challenge virus, the differences in the level of vaginal CpHV-1 genomes on day 1 post challenge could be due to the immune responses induced by the adjuvanted vaccine that could interfere with virus replication.

Clinical scores were also recorded after the vaginal challenge to monitor the clinical efficacy of the vaccine formulations (Fig. 4C). Goats vaccinated with inactivated CpHV-1 without adjuvant showed a significantly less severe clinical disease (characterized by milder lesions and faster healing) when compared to naïve animals that showed all the classical signs and kinetics of the herpetic disease [10]–[12], [23]. Challenge with virulent CpHV-1 did not produce any overt disease (that was evident upon visual inspection of the vaginas) in any goat vaccinated with inactivated CpHV-1 plus MF59™ (Fig. 4C). However, since histological analysis was not performed on CpHV-1-infected vaginal mucosae it is was not possible to exclude that the challenge virus had caused some pathological changes that were not visible upon gross examination of goats.

### Frequencies of CpHV-1-specific IFN-gamma producing cells in goats vaginally challenged with virulent CpHV-1

**Figure 5:**
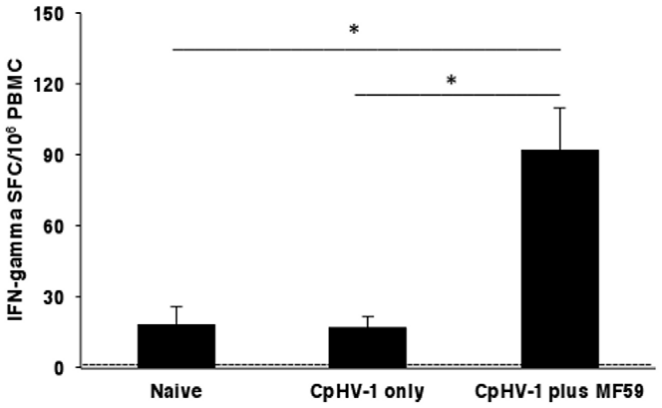
IFN-gamma SFC in PBMC isolated from goats subcutaneously vaccinated with inactivated CpHV-1 and challenged vaginally with virulent CpHV-1. Vaccines were given on day 0 and day 10. All goats were challenged vaginally two weeks following the second vaccination with 4 ml of virulent CpHV-1 suspension (10^5^ TCID_50_/50 µl) as described in Materials and Methods. Blood was drawn three weeks post-challenge and PBMC were isolated and stimulated *in vitro* as described in Materials and Methods. Frequencies of IFN-gamma Spot Forming Cells (SFC) were measured by ELISPOT. Data (arithmetic mean ± SD) are cumulative from 4 independent experiments performed as described in Materials and Methods with a total of 8 goats in the naïve group, a total of 8 goats in the inactivated CpHV-1 only vaccinated group and a total of 12 goats in the inactivated CpHV-1 plus MF59™ vaccinated group. A single asterisk (*) denotes probability, p, <0.05 that the IFN-gamma SFC in goats vaccinated with inactivated CpHV-1 plus MF59™ were equivalent to those of naïve goats. The same probability, p, <0.05 was observed when IFN-gamma SFC in goats vaccinated with inactivated CpHV-1 plus MF59™ were compared to those of goats vaccinated with inactivated CpHV-1 only. Statistical differences were calculated by the one-way ANOVA test followed by the Tukey's post-hoc test.

To determine the effect of the experimental infection on IFN-gamma production, PBMC were isolated from all challenged goats three weeks post-challenge and frequencies of IFN-gamma secreting cells were measured after CpHV-1 *in vitro* stimulation. As depicted in Figure 5, IFN-gamma producing cells were detected at similar levels in goats vaccinated with inactivated CpHV-1 only (17±5 IFN-gamma SFC/10^6^ PBMC) and in naïve goats (18±8 IFN-gamma SFC/10^6^ PBMC) while the frequencies of IFN-gamma producing cells in goats vaccinated with inactivated CpHV-1 plus MF59™ (92±18 IFN-gamma SFC/10^6^ PBMC) were significantly higher (an average 5-fold higher) than those observed in naïve goats or in goats vaccinated with inactivated CpHV-1 only. In addition, the frequency of IFN-gamma producing cells remained stable before and after vaginal challenge in goats vaccinated with inactivated CpHV-1 plus MF59™ (Fig. 3B and Fig. 5).

### Subcutaneous vaccination with inactivated CpHV-1 plus MF59™ interferes with the establishment of latency after vaginal challenge

**Figure 6:**
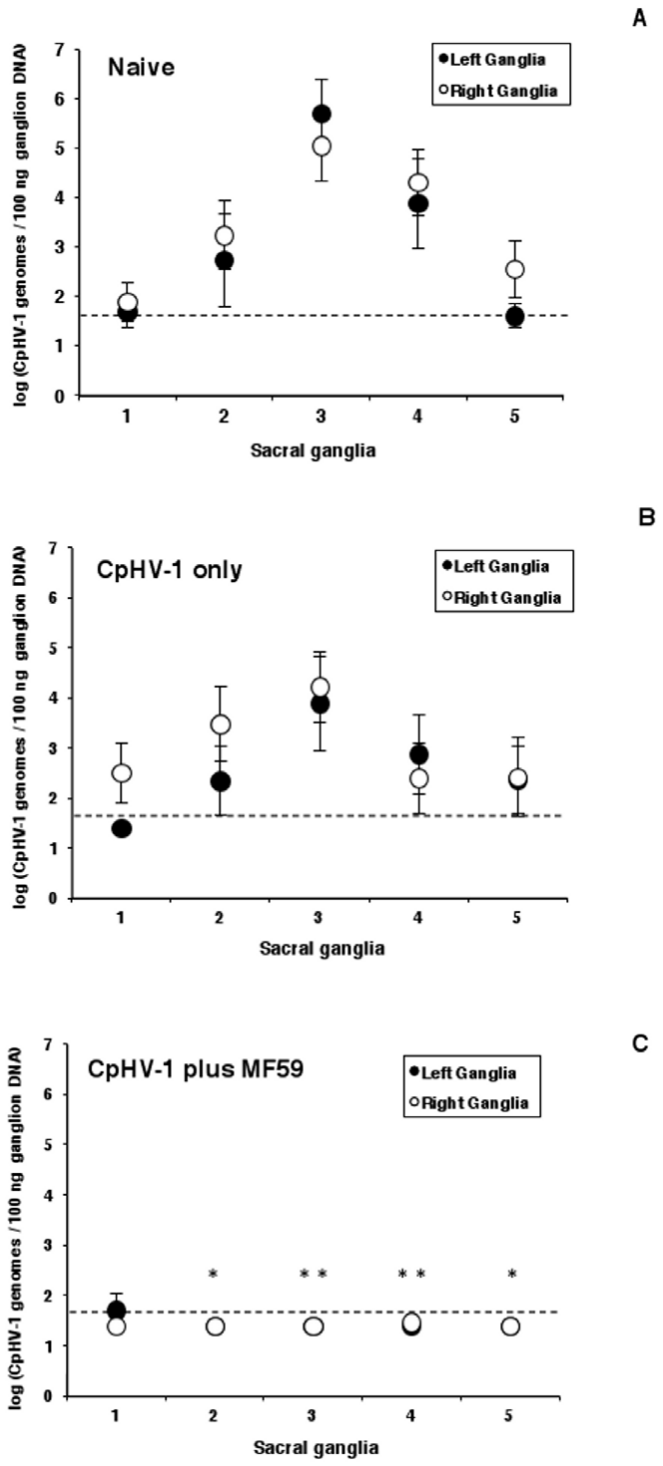
Distribution and quantitation of CpHV-1 genomic DNA in sacral ganglia from goats vaccinated with inactivated CpHV-1 and challenged with virulent CpHV-1. Vaccines were given on day 0 and day 10. All goats were challenged vaginally two weeks following the second vaccination with 4 ml of virulent CpHV-1 suspension (10^5^ TCID_50_/50 µl) as described in Materials and Methods. The five pairs of sacral ganglia were excised one month after challenge, the DNA was extracted from individual ganglion to detect and quantitate the number of CpHV-1 genomes by real-time PCR. Data (arithmetic mean ± SEM) are cumulative from 2 independent experiments i.e., experiment n.3 and experiment n.4, performed as described in Materials and Methods with a total of 6 goats in the naïve group, a total of 6 goats in the inactivated CpHV-1 only vaccinated group and a total of 6 goats in the inactivated CpHV-1 plus MF59™ vaccinated group. White circles (right ganglia), black circles (left ganglia). A single asterisk (*) denotes probability, p, <0.05 that the number of CpHV-1 genomes in the second (or in the fifth) pair of ganglia of goats vaccinated with inactivated CpHV-1 plus MF59™ was equivalent to that of naïve goats. A double asterisk (**) denotes probability, p, <0.001 that the number of CpHV-1 genomes in the third (or in fourth) pair of ganglia of goats vaccinated with inactivated CpHV-1 plus MF59™ was equivalent to that of naïve goats. Statistical differences were calculated by the one-way ANOVA test followed by the Tukey's post-hoc test.

To determine whether the inactivated CpHV-1 plus MF59™ vaccine influenced the establishment of latency, sacral ganglia were isolated from all challenged goats and subjected to a real-time PCR assay to detect and quantitate the latent CpHV-1 load. As reported previously [23], CpHV-1 DNA was variably detected in all five pairs of sacral ganglia isolated from naïve goats and a similar trend was observed in goats vaccinated with inactivated CpHV-1 only (Fig. 6A and 6B) thereby confirming that the amount and distribution of latent CpHV-1 in sacral ganglia do not appear to be related to the severity of the clinical signs of disease following challenge [23].

In naïve goats, the latent CpHV-1 genomes were most abundantly found in the second, third and fourth pair of sacral ganglia and they were approximately 1,500 times, 1,000,000 times, and 3,500 times above the limit of detection of the real-time PCR assay for the second, the third and the fourth ganglia, respectively (log_10_ 1.8±0.8 for the first pair; log_10_ 3.0±1.8 for the second pair; log_10_ 5.4±1.5 for the third pair; log_10_ 4.1±1.7 for the fourth pair; log_10_ 2.1±1.1 for the fifth pair). In addition, in naïve goats, CpHV-1 genomes were detected in 3 out of 12 first sacral ganglia (the average number of CpHV-1 genomes in these 3 ganglia was 40 times above the limit of detection of the real-time PCR assay) and in 4 out of 12 fifth sacral ganglia (the average number of CpHV-1 genomes in these 4 ganglia was 100 times above the limit of detection of the real-time PCR assay).

It was interesting to note that, in goats vaccinated with inactivated CpHV-1 plus MF59™, the CpHV-1 genome was undetectable in 58 out of 60 sacral ganglia examined and in particular it was undetectable in: i) 11 out 12 first sacral ganglia (with a number of CpHV-1 genomes in the latently infected ganglion being 35 times above the limit of detection of the real-time PCR assay); ii) 12 out of 12 second sacral ganglia; iii) 12 out of 12 third sacral ganglia; iv) 11 out of 12 fourth sacral ganglia (with a number of CpHV-1 genomes in the latently infected ganglion being less than 2 times above the limit of detection of the real-time PCR assay); v) 12 out of 12 fifth sacral ganglia. In goats vaccinated with inactivated CpHV-1 plus MF59™, the number of latent CpHV-1 genomes in the second, third, fourth and fifth pair of sacral ganglia was significantly different from that of naïve goats. Indeed, in four out of six goats vaccinated with CpHV-1 plus MF59™, the CpHV-1 genome was undetectable in all five pairs of sacral ganglia while the remaining two goats had detectable latent CpHV-1 DNA in one single ganglion (one goat in the first left ganglion and one goat in the fourth right ganglion).

## Discussion

In this study subcutaneous administration of a whole inactivated CpHV-1 vaccine plus MF59™ was able to confer effective protection against vaginal CpHV-1 challenge in goats. The vaccine-induced immune responses were protective as they reduced the replication of challenge virus in the genital mucosa to undetectable levels and significantly reduced the establishment of latency in sacral ganglia after challenge. Since the latent viral load is the most relevant factor that predicts reactivation rates of animals latently infected with HSV [30], [31], the present results suggest that vaccination with inactivated CpHV-1 plus MF59™ could at least influence the rate of recurrent infections thereby reducing the transmission of the infection to other animals in the same flock. To our knowledge this is the first study showing that a similar level of protection against an alphaherpesvirus could be achieved in a natural host by vaccination.

MF59™ is a detergent-stabilized oil-in-water emulsion consisting of small drops of oil (squalene) surrounded by a monolayer of non-ionic detergents. [14]. MF59™ is able to increase the immunogenicity of several types of antigens, promotes both antibody and cell-mediated immune responses and has an excellent safety record [14]–[17], [32]; it is indeed licensed for human use in the European Union and it is a component of seasonal and pandemic injectable human flu-vaccines [33]. The adjuvant MF59™ has been administered, mostly by the intramuscular route, to several animal species (from rodents to non-human primates) though data in ruminants are limited to immunogenicity not to vaccine efficacy [34]. After intramuscular delivery, muscle cells were shown to be targets of MF59™ [35], [36], and its adjuvant activity could be associated with the active recruitment of antigen presenting cells to the injection site. The high efficacy of MF59™ observed in the present study following subcutaneous administration with inactivated CpHV-1 could be due to the crucial role played by epidermal and dermal dendritic cells in priming naïve T cells [37]–[40]. In addition, the use of a whole virus as immunogen, rather than single glycoproteins, could have contributed to the observed high efficacy due to the presence of natural TLR ligands in the vaccine. In this regard, human trials have shown that HSV-2 subunit vaccines (containing gB and gD) are unable to confer full protection in humans [41]–[43]. However, it should be underlined that although, in the veterinary field, the use of a classical whole inactivated virus as immunogen could be more practical and less expensive than the use of individual antigens, further studies are mandatory in order to identify potential protective CpHV-1 antigens that could be tested for immunogenicity and efficacy in vaccination protocols.

The present study suggests that IgG produced following parenteral immunization of goats could transudate from serum to vaginal secretions contributing to protective immunity against vaginal CpHV-1 infection. The presence of Ag-specific IgG in the preferential site of entry of CpHV-1 would provide a first line of defense against infection that could be further implemented by the physiologic increase in serum transudation which occurs during the vaginal herpetic infection [44], [45]. On the other hand, CpHV-1-specific serum IgG and VN antibodies could contribute to protection by intercepting the virus escaped from the mucosa. Indeed, it is remarkable that serum antibodies specific to HSV have been shown to: i) neutralize herpesviruses at the axon terminus/synapses; ii) promote intra-axonal neutralization; iii) inhibit axonal spread to epidermal cells [46], [47]. Consistent with the above findings, the inactivated CpHV-1 plus MF59™ vaccine was able to interfere with the replication of CpHV-1 in the genital mucosa and to significantly reduce the load of latent CpHV-1 in sacral ganglia. Indeed, in some animals (i.e., 4 out of 6) immunized with inactivated CpHV-1 plus MF59™, the vaccine was able to reduce the establishment of latency to undetectable levels in all five pairs of sacral ganglia.

Several studies on immunity to HSV-1/HSV-2 vaccines [42], [43], [48]–[51] as well as studies on immunity elicited by inactivated whole MCMV vaccines [52]–[54] have shown that antigen-specific antibody responses produced after immunization are only able to limit the severity of the disease caused by these herpesviruses and that T cell responses play a major role in controlling primary infection and reactivations. Results from the present study seem to extend this observation also to CpHV-1. Indeed, immune serum antibodies generated following immunization seem to provide only partial protection from genital disease (e.g, as it occurs in goats vaccinated with inactivated CpHV-1 only), while appear to leave unabated acute viral replication in the genital mucosa thereby allowing the establishment of a latent infection in sacral ganglia. The data reported here suggest that, in goats vaccinated with inactivated CpHV-1 plus MF59™, CpHV-1-specific vaginal IgG and IFN-gamma production are associated with protective immunity against vaginal CpHV-1 infection although the relative contribution of antibody (mucosal and/or systemic) and IFN-gamma production to protection against vaginal CpHV-1 challenge remains to be specifically addressed in future studies.

The adjuvant MF59™ was able to induce high levels of antigen-specific IFN-gamma which is a key soluble mediator in control and resolution of HSV-2 infection [55]–[59]. Although, IFN-gamma is a major cytokine produced by T helper 1 lymphocytes and it induces CD8^+^ CTL, future studies will be necessary to determine the contribution of each PBMC subset (e.g., CD4^+^, CD8^+^, WC1^+^, NK) to IFN-gamma production in goats vaccinated with inactivated CpHV-1 plus MF59™. In addition, the analysis should be expanded to other cytokines and chemokines to test whether other soluble mediators contribute to the protection observed in goats vaccinated with inactivated CpHV-1 plus MF59™.

Two caveats of the current study are that: i) challenge was perfomed only two weeks following the second immunization; ii) only female goats were vaccinated. Future studies should employ larger numbers of animals of both sexes and longer time intervals should be allowed between vaccination and challenge. These studies would determine if the protective effect elicited by the inactivated CpHV-1 plus MF59™ vaccine persists for longer periods and could prove if the vaccine is also effective in males.

In conclusion, this is the first study showing that MF59™ in goats: i) provides effective adjuvant activity when administered subcutaneously with an inactivated CpHV-1 vaccine; ii) induces high levels of IFN-gamma by vaccine-specific PBMC as it has been reported in humans [60]

In perspective, due to the excellent safety and efficacy record of MF59™, the current study could help design new vaccines for pets and horses where safety and reactogenicity are primary concerns [61], [62] and for whom effective vaccines against some infectious diseases are needed (e.g, Feline Leukemia Virus, Feline and Canine Coronavirus, Canine and Equine Herpesvirus).
